# Evaluation of the relationship between the systemic inflammatory response and cancer-specific survival in patients with primary operable breast cancer

**DOI:** 10.1038/sj.bjc.6603682

**Published:** 2007-03-20

**Authors:** A M Al Murri, C Wilson, A Lannigan, J C Doughty, W J Angerson, C S McArdle, D C McMillan

**Affiliations:** 1 Royal Infirmary, University Department of Surgery, Glasgow, UK; 2 Western Infirmary, University Department of Surgery, Glasgow, UK; 3 Department of Surgery, Wishaw General Hospital, Lanarkshire, UK

**Keywords:** systemic inflammatory response, C-reactive protein, albumin, primary breast cancer, survival

## Abstract

The relationship between the systemic inflammatory response (as evidenced by elevated C-reactive protein and lowered albumin concentrations), clinico-pathologic status and relapse-free, cancer-specific and overall survival was examined in patients with invasive primary operable breast cancer (*n*=300). The median follow-up of the survivors was 46 months. During this period, 37 patients relapsed and 25 died of their cancer. On multivariate analysis, only tumour size (*P*<0.05), albumin (*P*<0.01) and systemic treatment (*P*<0.0001) were significant independent predictors of relapse-free, cancer-specific and overall survival. Lower serum albumin concentrations (⩽43 g l^−1^) were associated with deprivation (*P*<0.05), hormonal receptor negative tumours (*P*<0.01) and significantly poorer 3-year relapse-free (85 *vs* 93%, *P*=0.001) cancer-specific (87 *vs* 97%, *P*<0.0001) and overall survival (84 *vs* 94%, *P*=0.001) rates. The results of the present study suggest that lower preoperative albumin concentrations, but not elevated C-reactive protein concentrations, predict relapse-free, cancer-specific and overall survival, independent of clinico-pathologic status and treatment in patients undergoing potentially curative surgery for primary operable breast cancer.

Breast cancer is the commonest female malignancy and is a major cause of morbidity and mortality in the Western World. For example, in the UK, there are approximately 42 000 new patients each year and almost 13 000 deaths attributable to the disease (CancerStats, 2003).

It is increasingly recognised that variations in outcome in cancer patients are not solely determined by the characteristics of the tumour but also by the host-response factors (Balkwill and Mantovani, 2001; Coussens and Werb, 2002). However, the tumour–host interaction is complex and has yet to be fully understood. It is now accepted that the host systemic inflammatory response can be assessed by examining the changes in the concentrations of acute phase proteins, such as elevated circulating concentrations of C-reactive protein and low concentrations of albumin (Gabay and Kushner, 1999). It is of interest that, either singly or combined, these factors have been shown to be stage independent prognostic factors in patients with a variety of advanced cancers (McMillan *et al*, 2001; Mahmoud and Rivera, 2002; Forrest *et al*, 2003; Crumley *et al*, 2006a, 2006b).

There is also some evidence that the systemic inflammatory response has prognostic value in patients with metastatic breast cancer. For example, previous studies have reported that the presence of elevated circulating concentrations of C-reactive protein (Williams *et al*, 1990; Albuquerque *et al*, 1995; Zhang and Adachi, 1999; Al Murri *et al*, 2006) and low concentrations of albumin (Heys *et al*, 1998; Al Murri *et al*, 2006) are associated with poorer survival.

However, few studies have examined the prognostic value of the systemic inflammatory response in patients with primary breast cancer (Mortensen and Rudczynski, 1982; Lis *et al*, 2003). Those studies that have, appear to have produced conflicting results with regard to whether C-reactive protein or albumin concentrations have independent prognostic value. For example, Mortensen and Rudczynski (1982) studied almost 300 patients with a follow-up period of 3–48 months post-surgery and reported that the presence of an elevated C-reactive protein concentrations was not an independent prognostic factor. In contrast, Lis *et al* (2003) reported that in almost 200 patients with a follow-up period of 6–84 months, albumin was a stage independent prognostic factor. However, in both these studies, median follow-up was limited and approximately 20% of patients studied had advanced disease.

Therefore, the prognostic value of C-reactive protein and albumin in patients with primary operable breast cancer remains unclear. The aim of the present study was to examine the relationship between clinico-pathologic status, C-reactive protein and albumin concentrations, measured before surgery and cancer-specific survival in patients with invasive primary operable breast cancer.

## PATIENTS AND METHODS

Three hundred patients presenting with invasive primary operable breast cancer to two hospitals (Western Infirmary, Glasgow and Wishaw General Hospital, Lanarkshire) in the West of Scotland between June 2001 and July 2003 were prospectively included in the study.

The extent of deprivation was derived from the 1991 census, using the postcode of residence at diagnosis (Carstairs and Morris, 1990, 1991). The results are presented by amalgamating the seven categories into three groups: affluent (categories 1 and 2), intermediate (categories 3–5) and deprived (categories 6 and 7).

Clinico-pathological data including age, deprivation category, histological type, tumour size and grade, lymph node status, oestrogen (ER) and progesterone receptor status. The type of surgery and the use of adjuvant treatment (chemotherapy, hormonal therapy and radiotherapy) was recorded.

Routine preoperative laboratory measurement of C-reactive protein, albumin and white cell count were carried out. At this time, no patients showed clinical evidence of infection or other active chronic inflammatory conditions such as rheumatoid arthritis or crohn's disease. The coefficient of variation for these measurements was less than 10%, as established by routine quality control procedures. The limit of detection of C-reactive protein concentration assay was 6 mg l ^−1^, with the upper limit of normal values being ⩽10 mg l^−1^.

The study was approved by the local Research Ethics committees.

### Statistics

As appropriate, data are presented as median and range, and comparisons between patient groups were carried out using the *χ*^2^ test or Mann–Whitney *U*-test. Statistical analysis was based on the seven individual deprivation categories. Grouping of the laboratory variables was carried out using standard thresholds (Goldwasser and Feldman, 1997; O'Gorman *et al*, 2000; McMillan *et al*, 2001; Maltoni *et al*, 2005).

The correlation between C-reactive protein, white cell counts and albumin concentrations was performed using the Spearman's Rank correlation. Relapse-free, cancer-specific and overall survival analyses of the group variables were performed using the Cox proportional hazard model. Deaths up to the end of May 2006 were included in the analysis. Multivariate survival analyses, including all covariates that were significant on univariate analysis, were performed using a stepwise backward procedure to derive a final model of the variables that had a significant independent relationship with survival. To remove a variable from the model, the corresponding *P*-value had to be greater than 0.10. Analysis was performed using SPSS software version 13.0 (SPSS Inc., Chicago, IL, USA).

## RESULTS

The baseline clinico-pathological characteristics of the patients with primary operable breast cancer (*n*=300) are shown in Table 1. Two hundred and thirty-three patients (78%) patients were over 50 years of age; 82 (27%) were in the most deprived categories 6 and 7.

Of the 300 patients, 254 (85%) patients had ductal carcinoma, 122 (41%) had a tumour larger than 2 cm and 130 (43%) had axillary lymph node involvement. The majority of patients had tumour grade II/III (81%) disease. Sixty-two (21%) had ER-negative tumours. Twenty out of 300 (7%) patients had evidence of pre-existing comorbidity, such as liver dysfunction, cardiovascular disease or diabetes mellitus. In one patient, this was severe enough to interfere with planned adjuvant treatment. In all, 288 (96%) patients received adjuvant treatment in the form of endocrine therapy and/or chemotherapy.

The majority had a white cell count, albumin and C-reactive protein concentrations in the normal range (96, 100 and 88% respectively) before surgery. White cell count was correlated with C-reactive protein (*r*_s_=0.13, *P*=0.023) but not albumin concentration (*r*_s_=0.03, *P*=0.587). Albumin was correlated with C-reactive protein concentration (*r*_s_=−0.19, *P*=0.002).

The minimum follow-up was 35 months; the median follow-up of the survivors was 46 months. During this period, 37 patients relapsed and 25 died of their cancer; a further 14 patients died of intercurrent disease.

On univariate survival analysis, age (*P*<0.10), tumour type (*P*<0.10), tumour size (*P*<0.0001), grade (*P*<0.01), lymph node involvement (*P*<0.01), hormone receptor status (*P*<0.0001), albumin (*P*⩽0.001), loco-regional treatment (*P*<0.0001) and systemic treatment (*P*<0.0001) were significantly associated with relapse-free survival. On multivariate analysis of these significant covariates, age (HR 5.02, 95%CI 1.49–16.93, *P*=0.009), tumour size (HR 2.34, 95%CI 1.18–4.62, *P*=0.015), albumin (HR 3.65, 95%CI 1.71–7.78, *P*=0.001), loco-regional treatment (HR 2.56, 95%CI 1.17–5.59, *P*=0.019) and systemic treatment (HR 2.26, 95%CI 1.54–3.32, *P*<0.0001) were significant independent predictors of relapse-free survival.

On univariate survival analysis, tumour size (*P*<0.0001), grade (*P*<0.01), lymph node involvement (*P*′⩽0.001), hormone receptor status (*P*<0.0001), albumin (*P*<0.01), loco-regional treatment (*P*<0.0001) and systemic treatment (*P*<0.0001) were significantly associated with cancer-specific survival. On multivariate analysis of these significant covariates, tumour size (HR 2.53, 95%CI 1.15–5.55, *P*=0.021), albumin (HR 4.44, 95%CI 1.60–12.28, *P*=0.004), loco-regional treatment (HR 3.55, 95%CI 1.30–9.67, *P*=0.013) and systemic treatment (HR 2.67, 95%CI 1.56–4.57, *P*<0.0001) were significant independent predictors of cancer-specific survival.

On univariate survival analysis, age (*P*<0.10), tumour size (*P*<0.0001), grade (*P*<0.05), lymph node involvement (*P*<0.05), hormone receptor status (*P*<0.01), albumin (*P*⩽0.001), loco-regional treatment (*P*<0.01) and systemic treatment (*P*<0.001) were significantly associated with overall survival. On multivariate analysis of these significant covariates, age (HR 4.19, 95%CI 1.26–13.91, *P*=0.019), albumin (HR 3.33, 95%CI 1.60–6.90, *P*=0.001), tumour size (HR 2.48, 95%CI 1.36–4.55, *P*=0.003) and systemic treatment (HR 2.10, 95%CI 1.45–3.05, *P*<0.0001) were significant independent predictors of overall survival.

Patients were then grouped according to albumin concentrations (>43/⩽43 g l^−1^) as shown in Table 2. A lower serum albumin concentration (⩽43 g l^−1^) was associated with deprivation (*P*<0.05), hormonal receptor negative tumours (*P*<0.01) and significantly poorer 3-year relapse-free (85 *vs* 93%, *P*=0.001), cancer-specific (87 *vs* 97%, *P*<0.0001) and overall survival (84 *vs* 94%, *P*=0.001) rates.

## DISCUSSION

Predicting recurrence and survival following potentially curative surgical resection for primary operable breast cancer is conventionally based on standard clinico-pathological criteria such as age, tumour size and grade, nodal status and hormonal receptor status. However, it was of interest that other host-related factors, such as the systemic inflammatory response, have previously been shown to be associated with poor survival following a potentially curative resection for a variety of cancers including gastro-oesophageal, pancreatic, colorectal and urinary bladder cancers (McMillan *et al*, 2003; Hilmy *et al*, 2005; Jamieson *et al*, 2005; Crumley *et al*, 2006a, 2006b).

In the present study, albumin but not C-reactive protein was a significant, stage independent, predictor of survival in patients with primary operable breast cancer. The relationship between albumin concentrations and outcome are therefore consistent with those previously reported in patients with advanced breast cancer (Heys *et al*, 1998; Al Murri *et al*, 2006).

Previous studies have shown that elevated C-reactive protein concentrations have prognostic value in patients with a variety of primary operable tumours (Ikeda *et al*, 2003; McMillan *et al*, 2003; Hilmy *et al*, 2005; Jamieson *et al*, 2005) and also in patients with advanced breast cancer (Williams *et al*, 1990; Albuquerque *et al*, 1995; Zhang and Adachi, 1999; Al Murri *et al*, 2006). It was therefore unexpected that C-reactive protein, either as a continuous or categorical variable, was not a significant prognostic factor in the present study.

The basis of this observation is not clear. However, an elevated C-reactive protein concentration (>10 mg l^−1^) was seen in only 12% of patients, a proportion lower than previously seen in both primary operable disease (approximately 20–40%) and in advanced cancer (approximately 40–80%). This may, in part, reflect the relatively small number of events relating to relapse-free survival, cancer-specific and overall survival in the present study.

It was of interest that, of the two acute-phase proteins examined in the present study, albumin, generally considered to be a relatively insensitive measure of the systemic inflammatory response, had independent prognostic value, even with values within the normal range. It has been previously shown that small reductions in albumin concentrations are associated with certain comorbid conditions such as liver dysfunction, cardiovascular disease or diabetes mellitus and poorer survival in the general population (Goldwasser and Feldman, 1997). In the present study, only 7% of patients had such comorbidity (only one patient had planned adjuvant treatment altered) and therefore this would suggest that there may be an interaction between breast cancer and albumin concentrations, which might impact on the survival of these patients.

The mechanism by which a low serum albumin might impact on relapse-free, cancer-specific and overall survival is not clear. It may reflect the biological functions of circulating albumin including binding and transporting of hormones and growth factors, inhibition of platelet function and thrombosis (Margarson and Soni, 1998). Furthermore, albumin in the breast cancer cell cytosol may inhibit tumour growth (Soreide *et al*, 1991) and tumour cell proliferation by modulating the activities of autocrine growth regulatory factors (Laursen *et al*, 1990).

In the present study, conventional prognostic factors, including tumour size, histological grade, nodal status and hormonal receptor status were significant on univariate analysis. However, including potentially curative loco-regional treatment and systemic treatment based on hormonal receptor status in the multivariate analysis, nodal status was not independently significant. This probably reflects the close association between nodal status and the treatment received, and their relative impact on relapse and survival.

Nevertheless, lower albumin concentrations before surgery, even those within the normal range, may be a sensitive measure of those patients likely to recur and therefore be used to identify high-risk patients and alter their treatment accordingly. Furthermore, a follow-up measure of albumin, after the systemic inflammatory response of treatment has resolved, may be of a value to establish whether treatment of the primary tumour has resulted in recovery of albumin concentrations (McMillan *et al*, 2003; Hilmy *et al*, 2005; Jamieson *et al*, 2005). Clearly, further studies are required to establish the prognostic value of preoperative albumin concentrations and to determine whether longitudinal albumin concentrations may also be of value.

In summary, the results of the present study suggest that preoperative albumin concentration predicts survival, independent of tumour-based factors, in patients undergoing potentially curative surgery for primary operable breast cancer.

## Figures and Tables

**Table 1:** The clinical and pathological characteristics of patients with invasive primary operable breast cancer and survival (relapse-free, cancer-specific and overall survival)

		**Relapse-free survival**		**Cancer-specific survival**		**Overall survival**	
	**Patients**	**Hazard ratio**	***P*-value**	**Hazard ratio**	***P*-value**	**Hazard ratio**	
	**(*n*=300)**	**(95% CI)**		**(95% CI)**		**(95% CI)**	***P*-value**
Age (⩽50/>50 years)	67/233	2.52 (0.89-7.11)	0.081	1.56 (0.54-4.54)	0.416	2.60 (0.93 – 7.33)	0.070
Deprivation (1–2/3–5/6–7)^*^	39/179/82	0.93 (0.75–1.14)	0.485	1.01 (0.79–1.31)	0.915	1.10 (0.90–1.36)	0.360
							
Type (special type/lobular/ductal)	11/35/254	3.09 (0.83–11.55)	0.094	14.97 (0.33–677.82)	0.164	1.23 (0.59–2.60)	0.583
Size (⩽20/21–50/>50 mm)	178/117/5	3.29 (1.83–5.94)	<0.0001	3.56 (1.74–7.26)	<0.0001	2.86 (1.62–5.06)	<0.0001
Grade (I/II/III)	56/148/95	2.23 (1.33–3.74)	0.002	2.85 (1.46–5.58)	0.002	1.74 (1.07–2.82)	0.026
Involved lymph node (0/1–3/>3)	169/92/38	1.90 (1.25–2.88)	0.003	2.37 (1.44–3.93)	0.001	1.52 (1.01–2.30)	0.046
Hormonal receptor status (ER+ PR+/ER+ PR− or unknown/ER− PR− or unknown)	116/122/62	2.61 (1.66–4.13)	<0.0001	2.76 (1.57–4.85)	<0.0001	1.94 (1.26–2.97)	0.002
							
White cell count (10^9^ l^−1^)^**^	7.0 (3.4–17.4)	0.97 (0.83–1.15)	0.755	0.94 (0.76–1.16)	0.559	1.04 (0.89–1.21)	0.619
White cell count (<8.5/8.5–11/>11 × 10^9^ l^−1^)	230/53/13	0.81 (0.41–1.60)	0.547	1.07 (0.52–2.21)	0.857	1.36 (0.81–2.29)	0.247
Albumin concentration (g l^−1^)^**^	44 (35–52)	0.88 (0.78–0.99)	0.035	0.82 (0.71–0.94)	0.006	0.88 (0.78–0.98)	0.026
Albumin concentration (>43/⩽43 g l^−1^)	155/114	3.39 (1.61–7.12)	0.001	5.01 (1.85–13.57)	0.002	3.23 (1.58–6.59)	0.001
C-reactive protein concentration (mg l^−1^)^**^	⩽6 (⩽6–66)	0.96 (0.88–1.04)	0.293	0.97 (0.89–1.06)	0.518	0.97 (0.90–1.04)	0.391
C-reactive protein (⩽10/>10 mg l^−1^)	265/35	0.40 (0.10–1.68)	0.211	0.62 (0.15–2.65)	0.522	0.60 (0.19–1.95)	0.395
Loco-regional treatment (mastectomy or conservation surgery+radiotherapy/mastectomy+radiotherapy)	221/79	3.70 (1.94–7.06)	<0.0001	5.51 (2.43–12.48)	<0.0001	2.40 (1.27–4.51)	0.007
Systemic treatment (ER-based treatment) (hormonal/hormonal+chemotherapy/chemotherapy/none)	150/86/52/9	2.03 (1.43–2.88)	<0.0001	2.59 (1.67–4.01)	<0.0001	1.93 (1.37–2.73)	<0.001

Abbreviations: CI, confidence interval; ER, oestrogen receptor; PR, progesterone receptor.

^*^Individual deprivation categories were used in the statistical analysis and ^**^median (range).

**Table 2:** The clinico-pathological characteristics of patients with invasive primary operable breast cancer according to albumin concentrations

	**Albumin >43 g l^−1^**	**Albumin ⩽43 g l^−1^**	
	**(*n*=155)**	**(*n*=114)**	***P*-value**
Age (⩽50/>50 years)	35/120	26/88	0.965
Deprivation (1–2/3–5/6–7)^*^	26/90/39	13/68/33	0.011
			
Type (special type/lobular/ductal)	8/17/130	3/15/96	0.524
Size (⩽20/21–50/>50 mm)	96/57/2	62/49/3	0.384
Grade (I/II/III)	31/72/52	18/65/30	0.201
Involved lymph node (0/1–3/>3)	93/47/15	60/35/18	0.269
Hormonal receptor status (ER+ PR+/ER+ PR− or unknown/ER− PR− or unknown)	80/47/28	35/54/25	0.002
			
White cell count (10^9^ l^−1^)^**^	6.8 (3.6–13.9)	7.0 (3.4–17.4)	0.920
White cell count (<8.5/8.5–11/>11 × 10^9^ l^−1^)	127/18/8	83/27/3	0.281
C- reactive protein (mg l^−1^)^**^	<6 (<6–57)	<6 (<6–66)	0.138
C- reactive protein (⩽10/>10 mg l^−1^)	136/19	102/12	0.660
			
Loco-regional treatment (mastectomy or conservation surgery+radiotherapy/mastectomy+radiotherapy)	120/35	78/36	0.098
Systemic treatment (ER-based treatment) (hormonal/hormonal+chemotherapy/chemotherapy/none)	74/52/25/3	64/25/21/4	0.185
3 years relapse-free survival rate^***^	93 (2)	85 (3)	0.001
3 years cancer-specific survival rate^***^	97 (1)	87 (3)	<0.0001
3 years overall survival rate^***^	94 (2)	84 (3)	0.001

Abbreviations: ER, oestrogen receptor; PR, progesterone receptor.

^*^Individual deprivation categories were used in the statistical analysis, ^**^median (range) and ^***^3-year survival rate (SE).
